# Transitional health care for patients with Hirschsprung disease and anorectal malformations

**DOI:** 10.1007/s10151-017-1656-2

**Published:** 2017-07-03

**Authors:** M. J. Witvliet, N. Petersen, E. Ekkerman, C. Sleeboom, E. van Heurn, A. F. W. van der Steeg

**Affiliations:** 1 Pediatric Surgical Center of Amsterdam, Emma Children’s Hospital AMC and VU University Medical Center, Boelelaan 1117, 1081 HV Amsterdam, The Netherlands; 2 Wilhelmina Children’s Hospital, Utrecht, The Netherlands; 3 Academic Medical Center, Amsterdam, The Netherlands; 4 Department of Medical Psychology, Center of Research on Psychology in Somatic Diseases (CoRPS), Tilburg University, Tilburg, The Netherlands

**Keywords:** Transitional health care, Hirschsprung disease, Anorectal malformation, Quality of life, Health-related quality of life

## Abstract

**Background:**

Hirschsprung disease (HD) and anorectal malformations (ARM) are congenital disorders with potentially lifelong consequences. Although follow-up is performed in most pediatric patients, transfer to adult health care is often problematic. This study assesses transitional care with the help of questionnaires in consultation with adult patients.

**Methods:**

This study was conducted in an outpatient clinic of a pediatric surgical center in the Netherlands. All patients born and treated for ARM or HD before 1992 were invited to visit our clinic. Patients completed questionnaires concerning disease-specific functioning and quality of life at an initial visit to in response to which individual treatment plans were modified. Patients were reviewed 1 year later.

**Results:**

Twenty-seven patients (17 ARM and 10 HD), mean age 27.9 years (range 17–64 years) of the 168 invited visited the transitional clinic (17%). Passive fecal incontinence was reported by 7/27, other defecatory problems, including urge incontinence and incomplete evacuation in 17/27 and anal or abdominal pain reported by 9/27. Quality of life was lower than a matched population. Only 13/27 returned for repeat assessment at 1 year; however, a further 8 reported that that their problems had resolved. In those attending follow-up, negative thoughts and feelings about their condition had decreased and one more patient was fully continent. There was no change in quality of life, bowel function or pain recorded. Twelve out of thirteen patients reported that they had found the transitional clinic satisfactory.

**Conclusions:**

The transitional outpatient clinic provides care adapted to the needs and wishes of adult HD and ARM patients. It is a novel addition to quality of care of patients with complex congenital disorders.

## Introduction

The incidence of anorectal malformations (ARM) and Hirschsprung disease (HD) is both approximately 1 in 5000 live births [1, 2]. In our hospital, every year approximately 10–15 children are born with either ARM or HD. ARM comprises a wide spectrum of anomalies of the anus and rectum, but is also often associated with anomalies of other tracts including the urinary and genital tract. In HD, there is a variable length of aganglionic colon, which is usually resected soon after birth. Patients with ARM or HD may experience mild-to-severe defecation disorders and other disease-specific problems throughout their entire life. Functional problems related to bowel function, the urinary tract system and sexuality are commonly associated with both diseases and may have a significant impact on quality of life (QoL) [3–6].

Most patients undergo periodic follow-up by the pediatric surgeon until the age of 18 years. Then, they are transferred to ‘adult-centered’ physicians. Unfortunately, in this transfer of care follow-up is often lost or discontinued [7, 8]. The incidence of both HD and ARM is relatively low, and therefore, expertise of adult-centered physicians in these specific disorders is difficult to maintain. It is increasingly recognized that continuing follow-up and transitional care of patients with complex problems, such as ARM or HD, is an essential part of health care [9]. Many authors have reported the need for a transitional outpatient clinic, or have described the need to define patients’ problems during the transition phase to facilitate the process of starting adult care [9–12].

However, to the best of our knowledge the effect of transitional health care for this group of patients has not been studied before and neither has the use of questionnaires in addition to a transitional clinic for patients with HD or ARM.

The aim of the study was to assess the impact of a transitional outpatient clinic and the use of disease-specific functioning and QoL questionnaires at an initial visit and after 1-year follow-up.

## Materials and methods

### Transitional outpatient clinic

The setup of this transitional outpatient clinic was based upon an earlier study performed in the Netherlands [5]. This study defined all important aspects of a transitional outpatient clinic, from a patient and caregiver point of view. The transitional clinic consisted of a network of specialists including one each of the following: dedicated pediatric surgeon, gastroenterologist, gastrointestinal surgeon, gynecologist, urologist, sexologist and a nurse specialized in stoma care and incontinence therapy (colorectal nurse practitioner). The pediatric surgeon was primarily responsible for the care and assessed the needs and problems of the patients. Patients, who required adult specialist treatment, were referred. Disorder-specific knowledge was transferred at the same time, preventing the repetition of time-consuming diagnostic procedures, which had already been performed during the patients’ childhood. Since this study focused on evaluation of healthcare practice, specific approval of the medical ethical board was waived.

### Patients

To evaluate the design of our transitional outpatient clinic, we invited all adult patients born before 1992, surgically treated for ARM or HD at our pediatric surgical center. If patients made an appointment for the clinic, they received a set of questionnaires and were asked to complete an informed consent form to allow us to use the questionnaires not only for direct patient care but also to evaluate the need of transitional care.

One year after this first evaluation, all patients who had visited the clinic were asked to complete a second set of questionnaires.

### Questionnaires

Demographics and disease-specific functioning were assessed using a questionnaire based on one designed by MacMillan et al. [13]. This questionnaire consists of 53 items during assessment 1 and 48 items during assessment 2. The items form 3 subscales (demographics, disease-specific functioning and psychosocial items) and have been translated using the international standard of translation and back-translation [14]. The response scale is a categorical. The English version of the MacMillan questionnaire has been validated, and its psychometric properties are good [13]. The Dutch version is not officially validated.

QoL was assessed using the Dutch version of the World Health Organization QoL assessment instrument (WHOQOL-100) [15]. This assessment instrument contains 100 items assessing QoL in 24 facets covering six domains (physical health, psychological health, level of independence, social relationships, environment and spirituality/personal beliefs) and a general evaluative facet (overall QoL and general health). In this study, we used the four-factor structure of the WHOQOL-100 that was described in earlier studies [16]. The response scale was a 5-point Likert scale. Scores on each facet and domain can range from 4 to 20. The reliability and validity of the instrument are good [17].

The third questionnaire was developed for the purpose of this study. This is a 20-item questionnaire, which was designed in order to evaluate patient experience with the transitional outpatient clinic. It focused on improvement in physical and emotional functioning due to changes in treatment and overall patient satisfaction (Table 1). Validity and reliability have not yet been tested.

**Table 1:** Transitional outpatient clinic evaluation questionnaire

1. How often have you visited the transitional outpatient clinic?
2. Which specialists have you visited after your visited the clinic?
3. Where you satisfied with the consult. If not, why not?
4. Where you on a bowel management program before you visited the clinic?
5. Did you start or adjust your bowel management program?
6. After starting or adjusting this bowel management. Have you experienced less complaints?
7. Have you started new medication after the visit to the clinic?
8. Do you feel that this medication helps you control your bowels, and that complaints become less?
9. Have you had any surgery as result of your visit to the clinic?
10. Has this surgery helped reducing your complaints?
11. Was the waiting time for the clinic long and a problem for you?
12. Did you feel that the specialist had enough time to talk to you and discuss all your problems during the visit?
13. Was it possible to see all needed specialists during the first visit? If no, was this of any inconvenience?
14. How do you want to stay in contact with the clinic?
15. Do you have contact with other patients?
16. If we would organize an event to talk about specific problems you and other patients experience (sexuality, bowel management, the disease itself) would you be interested?
17. You have completed several questionnaires before your visit. Were you satisfied with these questionnaires, and did it help you to define your problems?
18. Did you miss any questions?
19. Were you satisfied with the complete experience of the transitional outpatient clinic?
20. What could be adjusted in the transitional outpatient clinic?

The MacMillan and the WHOQOL-100 were used during the first assessment. The questionnaires were completed at home and sent back to the pediatric surgeon prior to the consultation.

One year after consultation, the MacMillan, the WHOQOL-100 and the transitional outpatient evaluation questionnaire were completed once more.

Clinical parameters were extracted from medical records.

### Statistical analysis

Statistical analysis was performed using descriptive statistics. Item scores of the WHOQOL-100 were deducted into facet and domain scores ranging from 4 to 20. Independent-sample *t* tests were used to measure the significance of observed differences in QoL between our sample groups and reference scores from the manual. The paired Student’s *t* test was used to measure the significance of differences in mean scores in QoL between the two measurements in time. The level of significance was raised to *p* < 0.01. Correlation analysis was performed according to Pearson’s method.

All analyses were performed with the Statistical Package for Social Sciences (SPSS version 23).

## Results

### Assessment 1

#### Patient characteristics

A total of 172 patients with ARM or HD, born between 1948 and 1992, were registered in the database of our hospital. Four patients had died, and therefore, a total of 168 patients (ARM *n* = 100 and HD *n* = 68) received an invitation to the transitional outpatient clinic. A total of 28 patients (17%) visited our clinic over the course of 2 years. One patient was mentally disabled and was not able to complete the questionnaires. His data were excluded from further analysis. The other 27 patients all participated in the study. Seventeen of these patients were diagnosed with ARM (7 women and 10 men) and 10 with HD (4 women and 6 men). The mean age of the patients was 27.9 years (range 17–64 years). Four of these 27 patients had an ostomy (14.8%), four patients (14.8%) used laxatives (*n* = 2) or anti-diarrheal drugs (*n* = 2) and seven (25.9%) used colonic irrigations as part of a bowel management program.

#### Referrals

The pediatric surgeon and the colorectal nurse practitioner informed the involved adult specialists about ARM and HD including the pathology of the disease, the operations that were performed and the possible (functional) problems during adolescence and adulthood, e.g., urinary, sexual problems and fecal incontinence. During the first consultation, problems were assessed by the pediatric surgeon and patients were referred to the appropriate adult specialist according to their specific problems (Table 2). All presenting problems could all be treated by the various specialists of the transitional team.

**Table 2:** Referral rate per specialist

Specialist	Number of patients referred
Urologist	2
Clinical geneticist	3
Gastrointestinal surgeon	4
Sexologist	1
Psychologist	2
Gynecologist	4
Plastic surgeon	2
Gastroenterologist	2

#### Physical disease-specific functioning

Seventeen patients (63%) reported problems with defecation (Table 3). These problems varied from incomplete evacuation of stool, urge incontinence or the need for manual pressure (pushing on the lower abdomen or next to the anus) to evacuate stool. In all but one case, these complaints were present for more than 6 months.

**Table 3:** Physical disease-specific functioning measured with MacMillan questionnaire (*n* = 27 patients)

Physical disease-specific functioning (MacMillan)		Number of patients (%)
Problems with stool	Yes	17 (63.0)
Duration of problems	From birth >6 months 1–6 months	1 (3.7) 15 (55.6) 1 (3.7)
Bowel management	Laxatives Anti-diarrheal Rinsing the bowel	2 (7.4) 2 (7.4) 7 (25.9)
Incontinence (minimally once a week)	Gas Mucus Thin fecal material Solid fecal material	21 (77.8) 9 (33.3) 10 (37.0) 7 (25.9)
Feeling that you need to pass a bowel motion	Yes	20 (74.1)
Hurrying to the toilet	Yes	16 (59.3)
Incontinence after having passed a bowel movement	Yes	10 (37.0)
Urine incontinence	Yes	5 (18.5)
Pain	Always Sometimes No	7 (25.9) 9 (33.3) 9 (33.2)

Seven patients (25.9%) reported weekly fecal incontinence for solid stool and 14 (51.9%) used a sanitary towel as protection against the loss of mucus, liquid or solid stool. Seven patients (25.9%) reported daily anal or abdominal pain, nine patients (33.3%) had occasional pain and nine patients (33.3%) experienced no pain. Data of two patients were missing. More functional results are found in Table 3.

#### Psychological disease-specific functioning

Five patients (18.5%) reported scheduling their daily activities around their defecation problems. Four (14.8%) did this occasionally. Fourteen patients (51.8%) were embarrassed because of their condition. Five patients (18.5%) reported depression, and 13 (48.1%) were worried that they smelt to others. Six patients (22.2%) were afraid to have sexual intercourse because of their condition, and 12 (44.4%) always checked for available toilets when entering an unfamiliar space. Nine patients (33.3%) were afraid to leave their house for social activities because of their condition.

#### Quality of life

Compared to reference scores from a healthy population, a trend was observed in which our patients scored lower on several QoL aspects. A significant difference was found for dependence on medication and treatment (*p* = 0.007) (Table 4). Furthermore, a positive effect was associated with better overall QoL and general health (*r* = 0.821; *p* < 0.001) and higher self-esteem (*r* = 0.824; *p* < 0.001). Self-esteem was positively correlated with overall QoL and general health (*r* = 0.782; *p* < 0.001).

**Table 4:** Quality of life measured with the WHOQOL-100

Variable	Mean score group 1 *n* = 27	St. dev group 1	Mean score ref.pop. *n* = 913	St. dev ref.pop.	*p* value
Pain and discomfort	8.5(+)	3.2	9.2	2.9	0.275
Energy and fatigue	13.3(−)	3.0	14.7	3.0	0.028
Sleep and rest	15.9(=)	3.0	15.9	3.2	0.169
Positive affect	15.4(+)	2.7	14.0	2.7	0.053
Think and learning	15.2(+)	2.0	14.9	2.6	0.416
Self-esteem	14.4(−)	3.1	14.7	2.9	0.623
Body image and appearance	14.3(−)	4.6	15.6	3.1	0.152
Negative affect	9.3(−)	3.8	9.4	3.2	0.862
Mobility	18.1(+)	2.8	17.3	3.0	0.147
Activities of daily living	16.2(−)	3.1	16.5	2.7	0.559
Dependence on medication or treatment	8.5(−)	4.1	6.2	3.0	**0.007**
Working capacity	17.0(+)	2.9	16.2	3.0	0.189
Personal relationships	16.4(+)	3.1	15.5	2.5	0.137
Social support	16.9(+)	3.5	14.6	3.2	**0.003**
Sexual activity	14.3(−)	4.8	14.8	3.4	0.614
Physical safety and security	17.1(+)	2.4	14.5	2.8	**<0.001**
Home environment	16.0(+)	2.6	14.8	3.4	0.019
Financial resources	16.1(+)	4.0	12.9	3.8	**<0.001**
Health and social care: access and quality	15.8(+)	2.7	13.8	2.9	**0.001**
Opportunity for acquiring info and skills	16.9(+)	2.3	14.4	2.9	**<0.001**
Participation and opportunity for recreation/leisure	16.2(+)	3.1	13.5	3.2	**<0.001**
Physical environment	15.6(+)	2.1	13.4	2.9	**<0.001**
Transportation	18.8(+)	2.1	15.1	3.6	**<0.001**
Overall QoL and general health	15.4(+)	2.8	14.9	2.9	0.325

A bold *p* value represents a level of significance of 0.01 or lower

Group 1: patient population (sample population); ref.pop: reference scores: international healthy population. Patient scores compared to reference scores indicated with + (higher) or − (lower) and level of significance (two-tailed)

### Assessment 2

#### Patient characteristics

Of the 27 patients who participated in the study, 13 patients (ARM *n* = 10, HD *n* = 3), seven males, mean age 32.2 (range 18–65) completed and returned the second questionnaire. Of the remaining 14, 8 patients did not want to participate because they experienced no ongoing problems, three because the clinic had not brought what they hoped for and three were lost to follow-up.

#### Disease-specific functioning (physical and psychological)

Of the 13 patients with two evaluations, five reported full continence at assessment 2, compared to four a year earlier. Answers to items concerning control of bowel function (e.g., urge, use of laxatives or pads) and pain were the same in both assessments.

Disease-specific negative thoughts and feelings had decreased, and in addition, patients experienced more fun in their lives since visiting the transitional outpatient clinic. Fear of engaging in sexual activity did not change (Table 5).

**Table 5:** Comparing disease-specific functioning (psychological) of patients that complete both assessments using the MacMillan questionnaire

Because of my condition, I feel or experience	Time interval one (*n*)*	Time interval two (*n*)*
Worried not to reach a toilet in time	Agree: 61.5% (13)	Agree: 30.8% (13)
Embarrassed	Agree: 76.9% (13)	Agree: 50.0% (12)
Worried about smelling	Agree: 69.2% (13)	Agree: 45.5% (11)
Unhealthy	Agree: 53.8% (13)	Agree: 8.3% (12)
Less fun in life	Agree: 30.8% (13)	Agree: 8.3% (12)
Less sexual activities	Agree: 23.1% (13)	Agree: 41.7% (12)
Different than others (peers)	Agree: 61.5% (13)	Agree: 50.0% (12)
Afraid to engage in sexual activities	Agree: 30.8% (13)	Agree: 33.3% (12)
Limited in dietary options	Agree: 30.8% (13)	Agree: 30.8% (13)
The need to always be in close proximity to a toilet to avoid incontinence problems	Agree: 30.8% (13)	Agree: 30.8% (13)
The need to organize my daily activities around my stool	Agree: 23.1% (13)	Agree: 15.4% (13)
Afraid to travel	Agree: 7.7% (13)	Agree: 0% (13)
Not in control of my bowel function	Agree: 69.2% (13)	Agree: 53.8% (13)
Worried about fecal incontinence	Agree: 38.5% (13)	Agree: 25.0% (12)
Less capable of doing the things I love	Agree: 15.4% (13)	Agree: 16.7% (12)
Depressed	Agree: 7.7% (13)	Agree: 8.3% (12)

* *n* = The total number of patients that answered this question

#### Quality of life

Positive changes in QoL over time were not observed in this group. An improvement in the pain and dependence on medication and treatment facets of the score were observed, but this improvement was not significant.

#### The experience of the transitional outpatient clinic

Twelve of the 13 patients, who visited the clinic and participated in the second survey, found that the transitional care they had received was satisfactory. Operative treatment was offered in two cases (ileostomy revision and rectal resection). Bowel management programs (colonic irrigation and/or enemas) were adjusted when required (*n* = 3, 23.1%). No changes in medication were made.

## Discussion

Transitional care is defined as a set of actions designed to ensure the coordination and continuity of health care and safe and timely transfer as patients transfer between different locations or different levels of care at the same or different institution [18, 19]. In order to improve the transition from pediatric surgical care to adult medical and surgical care, a transitional outpatient clinic was started in our hospital. The main goal of this outpatient clinic was to assist in the transition, transferring specific information and avoiding unnecessary investigations and operations. This transitional clinic will be used in the future in the transition phase from adolescent to adult-centered care. However, in order to get healthcare professionals familiar with the demands of these specific patient groups it was decided to pump prime the process with adult patients and to evaluate both the setup of the clinic and the use of questionnaires.

Only 17 percent of the invited patients attended our transitional clinic. This means at least 1 in 6 adult patients had complaints that they thought might improve with further treatment or had questions about their disease. The reasons for non-attendance and morbidity in the remaining 83% are not known. Most of those invited were not in active follow-up. Invitations were sent to the last known address, and therefore, some will have not reached the intended person. It may also be that chronic patients find it difficult to visit the hospital again, once they have learned to live with their handicap or problems.

Patients with ARM or HD may have problems with fecal continence, urinary continence and sexual function. Because of the socially undesirable character of many complaints, it was hypothesized that the use of validated questionnaires (disease specific functioning and QoL) would have additional value over a pediatric surgical consultation alone [20]. In individual cases, it was observed that complaints were not reported during face-to-face consultation, but became apparent when the questionnaires were completed away from healthcare professionals. Problems raised in questionnaires alone were addressed during subsequent consultation. Treatment not only focused on functional problems but also focused on the psychological aspects of problems. Questionnaires provided the healthcare professional with a structured overview of each patient’s overall functioning (physically, mentally and psychosocially) enabling ‘tailor-made’ personal care.

Fecal incontinence, control of bowel function and anal and/or abdominal pain caused by the condition were the areas of main concern within our patient group. This is in accordance with data from an earlier study evaluating disease-specific function in patients with ARM [3]. There was, however, a striking difference between this study and ours: In our group, a smaller number of patients used medication (e.g., laxatives) or bowel management programs. This is an important finding since bowel management programs have been shown to be effective [21, 22].

This study showed that overall QoL was not influenced by disease-specific function. Chronically ill patients often develop coping strategies, which result in good overall QoL scores despite evident impairment of physical and mental functioning [23]. However, in chronically ill patients specific facets of QoL can be worse than in the reference population. This could possibly be explained by the strong desire of chronically ill patients to be ‘normal’ [24]. In addition, patients tend to adjust to their situation, which results in a ‘response shift’ when using questionnaires [23]. In our patient group, for example, the facets participation in recreation/leisure, transportation and physical environment were scored significantly better than in the reference population.

This study also showed the influence of the impairment in psychosocial functioning on the patients’ self-esteem, with self-esteem having a major influence on overall QoL. A previous study showed that self-esteem can lead to better health and social behavior and low self-esteem can even be a risk factor for mental disorders and social problems [25].

Patients who visited the transitional outpatient clinic were satisfied overall with their consultation and the advice and treatment given to them. Eight patients did not want to participate in the follow-up study because their problems had resolved. From this it may be concluded that the transitional clinic is valuable for a substantial proportion of patients. Many patients in our study lived with their complaints for years before they visited our clinic. Starting this transitional care at 12–18 years of age will hopefully identify problems earlier and make the transition into adult care easier.

One of the limitations of this study was the small number of patients. It is questionable if this group of patients is representative of all patients operated on for ARM or HD.

Future studies should also focus on the needs and wishes of younger patients in transitional care [1, 2, 26]. Key points in transitional care for these patients would be disease education, sexual education and bowel management programs.

## Conclusions

The transitional outpatient clinic provides care adapted to the needs and wishes of adult HD and ARM patients. Prolonged follow-up and transition of care offer the opportunity to modify existing treatment and to diagnose new complaints that present at an older age. This is a novel addition to good quality care of patients with ARM and HD.

The use of questionnaires in the transitional outpatient clinic is of value in the evaluation of disease-specific functioning and individual facets of QoL. It provides the healthcare professional with a helpful tool to determine the complex interrelation between physical and emotional functioning of HD and ARM patients. Self-esteem greatly affects overall QoL. This should be assessed and may be the target of the overall treatment.
